# Cervical screening under wartime: resilience and quality of laboratory services in Ukraine during the Russian invasion

**DOI:** 10.3389/fpubh.2026.1909774

**Published:** 2026-08-11

**Authors:** Pavlina Botsun, Iryna Omelianenko, Yurii Sava, Mariia Panko, Larysa Natrus, Nazarii Kobyliak, Oksana Sulaieva

**Affiliations:** 1 Bogomolets National Medical University, Kyiv, Ukraine; 2 Medical Laboratory CSD, Kyiv, Ukraine; 3 Ukrainian Association for Precision Medicine, Kyiv, Ukraine

**Keywords:** Bethesda system, cervical screening, cytopathology quality, HPV 16, HPV 18, HPV testing, laboratory resilience, Ukraine

## Abstract

**Introduction:**

Since February 2022, Ukraine has been facing the challenges of active armed conflict, undermining its healthcare system. Despite the ongoing war, the national cervical cancer screening program was launched in 2025. However, little is known about whether laboratory diagnostic quality can be maintained under these conditions. This study aimed to assess the resilience of cervical cancer screening services in Ukraine during wartime by evaluating the temporal dynamics of PAP smears, HPV testing, and their quality indicators.

**Materials and methods:**

This single-center, repeated cross-sectional study included laboratory data collected on PAP smears from 946,451 cases and 114,974 HPV tests retrieved from the database of the Medical Laboratory CSD in 2021–2025. The Annual Bethesda System 2014 (BS 2014) category proportions and quality metrics (including the ASC/SIL ratio and non-diagnostic rate) were assessed and compared with the benchmarks from BS 2014 and the College of American Pathologists (CAP). HPV testing data included overall positivity rates and HPV 16/18 genotype distribution.

**Results:**

The volume of PAP tests decreased by 21.5% in 2022; however, there was a significant recovery in 2023, with an increase of 85.3% in 2025 compared to the pre-war baseline (2021). Each Bethesda category proportion for every year aligned within international benchmark ranges. The five-year CSD medians were as follows: NILM—91.27% (89.24–91.61%); ASC-US—4.45% (3.94–5.38%); LSIL—2.83% (2.49–3.57%); HSIL—0.38% (0.31–0.62%), non-diagnostic—0.80% (0.54–1.03%); SCC—0.024% (0.016–0.051%); and AGC—0.25% (0.22–0.26%). Quality metrics were consistent with global reference values, with a total abnormal sample rate of 7.81% (7.7–9.67%), ASC/SIL ratio of 1.36 (1.18–1.66), and ASC-US/LSIL ratio of 1.47 (1.28–1.79). HPV testing expanded at a disproportionately higher rate than Pap test utilization, surging 27.8-fold over the wartime period. HPV positivity declined significantly from 21.07% in 2021 to 17.16% in 2025 (*p* = 0.011). While HPV16 prevalence remained relatively stable, the relative contribution of HPV18 nearly doubled, rising from 5.6 to 10.4% among HPV-positive individuals (*p* = 0.008).

**Conclusion:**

Cytopathological reporting patterns remained within benchmark ranges under 4 years of armed conflict in Ukraine, with diagnostic performance of CSD LAB corresponding to international benchmarks. The rapid HPV testing expansion associated with an increased rate of testing among younger women, a decline in HPV positivity among the 30–65 cohort, and a rise in the HPV 18 proportional rate have direct implications for national vaccination and screening policies.

## Introduction

Cervical cancer remains a major preventable cancer, ranking as the fourth most common female malignancy globally, with approximately 662,044 new cases and 348,709 deaths recorded in 2022 (1). Persistent infection with high-risk oncogenic human papillomavirus (HPV) genotypes, especially with HPV16 and HPV18, is the essential etiological factor in the majority of cases of squamous cell carcinoma (SCC) and more than 70% of cervical adenocarcinomas (2). For more than six decades, screening programs based on the Papanicolaou (Pap) test have demonstrated effectiveness in reducing cervical cancer incidence and mortality by enabling the detection of precancerous lesions classified under the Bethesda System 2014 (BS 2014) terminology. Contemporary international guidelines, provided by the WHO, American Society for Colposcopy and Cervical Pathology (ASCCP), and ESGO, recommend the integration of validated high-risk HPV DNA testing as a primary approach for cervical screening alongside or followed by cytology (3).

Compared to the majority of European countries, Ukraine has historically faced a disproportionate burden of cervical cancer and operated an opportunistic, cytology-dominant screening model before 2022 (4).

Armed conflict is a well-documented cause of disruptions in preventive services: a systematic review of 20 studies across 8 conflicts found evidence that war increases cervical cancer incidence and mortality, primarily through infrastructure collapse, population displacement, and healthcare resource diversion (5). In addition to direct destruction, armed conflict imposes profound systemic pressures on laboratory and clinical services, including mass emigration of skilled healthcare workers, disruption of medical supply chains and cold-chain logistics, energy infrastructure damage, and continuous psychological burden on the retained staff. These factors can compromise diagnostic quality and operational continuity, even when physical facilities are not directly damage (6–8). The war in Ukraine has led to approximately 30,000 healthcare professionals enlisting in the Armed Forces or taking on voluntary roles, while more than 2,500 emigrated and over 4,500 have been internally displaced (9, 10). The primary barriers to healthcare service delivery were lack of staff (50%), infrastructure damage (35%), security concerns (34%), medical supplies (39%), and medical equipment (28%), which are further compounded by supply chain disruptions and power outage-related problems (11, 12). These challenges have worsened Ukraine’s historically low HPV vaccination coverage. Before 2022, HPV vaccination was recommended for girls aged 9–14 years but was not included in the national immunization program. As a result, Ukraine has one of the lowest HPV vaccine rates in Europe (13). Notably, in 2024, a national standard for cervical screening was implemented.

Whether cytopathology laboratories operating under sustained wartime conditions can maintain adherence to international quality standards and whether HPV testing can continue to expand alongside cytological screening remain an open question with direct implications for cervical cancer prevention policy in conflict-affected settings worldwide. The present study addresses this gap through a 5-year repeated cross-sectional analysis (2021–2025) of the complete screening dataset from CSD Laboratory, one of the largest private diagnostic laboratories in Ukraine.

This study aimed to assess the resilience and adaptive capacity of cervical cancer screening services in Ukraine during the wartime period by evaluating the temporal dynamics of PAP smears, HPV testing, and their quality indicators. During the study, we assessed: (1) annual trends in the volumes of Pap smears and HPV tests, (2) annual Bethesda category distributions compared to international benchmarks, (3) HPV positivity rates and HPV16/18 genotype dynamics, and (4) HPV-cytology concordance in a subset of the data.

## Materials and methods

### Study design and reporting

This study used a repeated cross-sectional design, analyzing data from CSD LAB, which operates across all Ukrainian regions. The data covered five consecutive calendar years from 2021 to 2025. The study is conducted in accordance with the STROBE checklist for cross-sectional design (14). Three contextual phases were defined *a priori*: (1) pre-war baseline (2021), (2) acute disruption (2022), and (3) recovery and expansion (2023–2025).

Data were retrieved from the electronic laboratory information system of the CSD laboratory. Following the February 2022 invasion, the central laboratory was temporarily relocated to Lviv and returned to Kyiv in May 2022 while maintaining continuous operations. Aggregated, de-identified annual summary data were extracted for the years 2021–2025. For subset analysis, case-level data for cytological-HPV correlation were extracted for the fourth quarter of 2025 (October–December 2025) (which included 98 abnormal cytology cases with documented HPV status). Ethical approval was granted by the CSD Laboratory Institutional Ethics Committee (protocol number IRB-2025-11/04, issued November 12, 2025). Given the retrospective nature of the study design and the use of historical database records, a formal waiver of written informed consent was explicitly approved by the Ethics Committee, in full accordance with national legislation and institutional requirements. No individual-level or identifiable patient records were accessible to the research team, and all data were analyzed as deidentified aggregates.

To address the strong relationship between patient age and cervical screening outcomes, the study population was stratified into four clinically distinct age cohorts: under 25 years, 25–29 years, 30–65 years, and over 65 years. These cohorts were designated to align with the chronological risk profiles of HPV infection dynamics. Annual demographic tracking was performed across the 5-year observation period (2021–2025) to evaluate shifts in the age distribution of the screened population. This demographic stratification was utilized to contextualize and interpret longitudinal variation in overall high-risk HPV positivity rates.

### Cytological and HPV testing analysis

All Pap smears were classified by qualified cytopathologists using BS 2014 terminology (15). Atypical glandular cells (AGC) subcategories including AGC-NOS (not otherwise specified) and AGC-FN (favored neoplastic) were consolidated into a single AGC group for trend analysis. Adenocarcinomas (AC) subcategories (endocervical, endometrial, NOS) were also consolidated into a single AC group. Endometrial cells in women ≥45 years (Em ≥ 45) were excluded from neoplastic analyses per BS 2014 criteria. Unsatisfactory specimens were reported separately, and their shares were presented as a percentage of the total number.

HPV testing was performed using the Cobas® HPV test (Roche Molecular Systems), an FDA-approved real-time PCR assay genotyping HPV16 and HPV18 individually and 12 other high risk oncogenic types (31, 33, 35, 39, 45, 51, 52, 56, 58, 59, 66, 68) as a combined ‘other high risk HPV’ result, using Cobas 6800 and Cobas 5800 (Roche).

### Outcome measures and quality indicators

Primary outcomes were annual Pap test and HPV test volumes, with Pap-to-HPV ratio calculation. Secondary outcomes included: annual Bethesda category proportions (% of informative specimens); the overall epithelial cell abnormality rate (≥ASC-US); ASC/SIL ratio [(ASC-US + ASC-H)/(LSIL + HSIL)], CAP benchmark ≤3.0, non-satisfactory rate, HPV positivity, HPV16, and HPV18 proportional shares among positive specimens.

### Statistical analysis

Statistical processing of the data was carried out using SPSS software, version 20.0 (SPSS Inc., Chicago, IL, USA), and GraphPad Prism, version 11.0 (GraphPad Software, La Jolla, CA, USA). Annual proportions were expressed as percentages with exact 95% Wilson confidence intervals (CI). Chi-square (*χ*^2^) test was applied to compare proportions between specific year pairs (e.g., 2021 vs. each wartime year). Two-sided *p* < 0.05 was considered statistically significant. The comparison with international benchmarks was based on the published median and range values from CAP interlaboratory program surveys (16) and large published series (17, 18).

## Results

### The trends in cervical cancer screening during wartime, 2021–2025

Over the 5-year observation period, CSD LAB processed a total of 946,451 Pap tests and 114,974 HPV tests (Table 1). In the pre-war baseline year (2021), 145,262 Pap tests were performed, though the count of HPV tests was much lower (1452), demonstrating the prevalence of cytological screening. Following the onset of hostilities in February 2022, Pap test volume dropped to 114,051, demonstrating a significant decline of 21.5% at the annual level (*p* < 0.001). At the same time, HPV testing grew, reaching 10,187 HPV assays in 2022, demonstrating a 7.0-fold increase over 2021. Such a shift led to the reduction of the Pap-to-HPV ratio from 100:1 to 11.2:1.

**Table 1:** Annual cervical screening volumes and Pap-to-HPV test ratios, CSD laboratory, Ukraine, 2021–2025.

Year	Pap tests (*n*)	Pap vs. 2021 (Δ%)	HPV tests (*n*)	HPV vs. 2021 (Δ×)	Pap-to-HPV ratio
2021 (pre-war)	145.262	Reference	1.452	Reference	100.0:1
2022	114.051	−21.5%	10.187	×7.0	11.2:1
2023	181.709	+25.1%	24.210	×16.7	7.5:1
2024	236.268	+62.6%	38.762	×26.7	6.1:1
2025	269.161	+85.3%	40.363	×27.8	6.7:1
Total 2021–2025	946.451	—	114.974	—	—

Pap-to-HPV ratio = annual Pap test count/annual HPV test count. Year-on-year Pap change calculated relative to 2021 baseline. × = ratio of annual HPV count to 2021 HPV count (*n* = 1,452).

**Figure 1:**
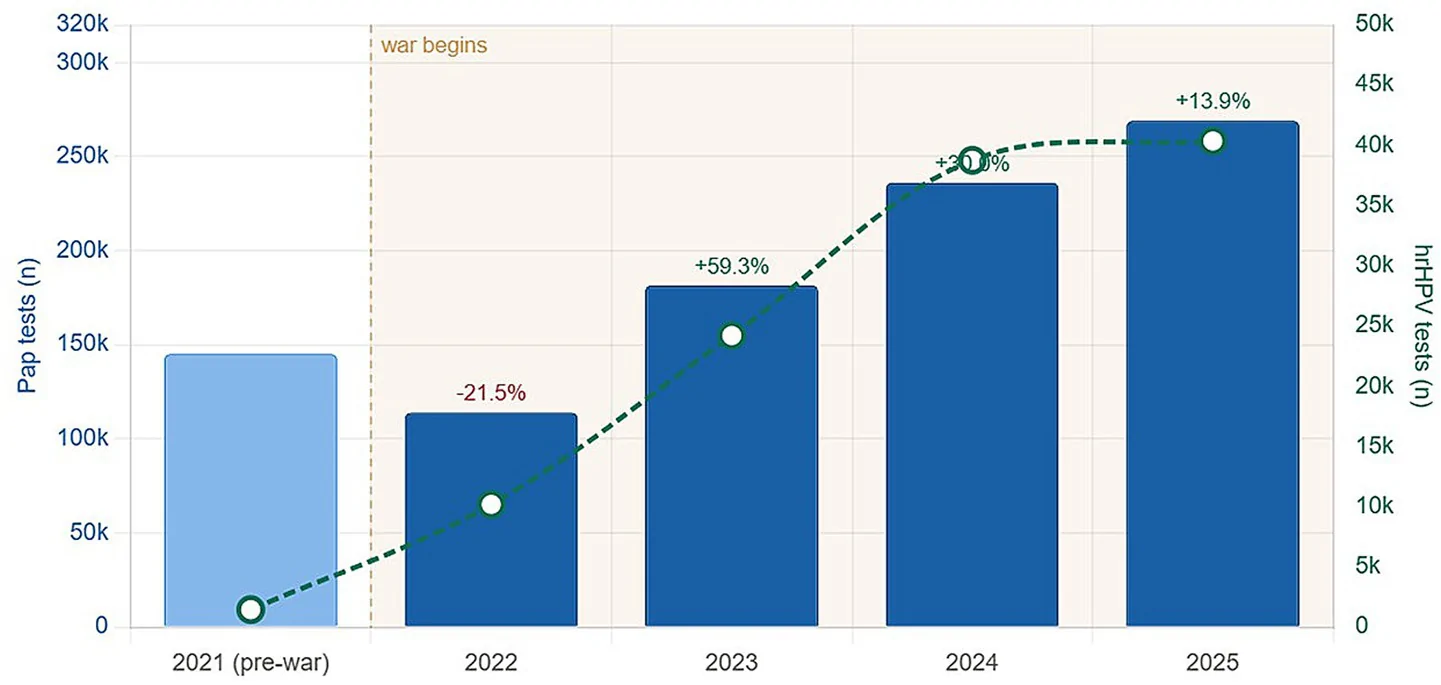
Temporal dynamics of PAP and HPV testing in Ukraine.

From 2023 onward, Pap test volumes recovered and exceeded pre-war levels reaching 181,709 in 2023 (+25.1% vs. 2021), with even more continuous elevations in 2024 (236,268: +62.6% vs. 2021) and 2025 (269,161; +85.3% vs. 2021; +13.9% vs. 2024). Similarly, HPV testing continued to expand, reaching 40,363 in 2025, which presents a 27.8-fold increase over the 5-year period. As a result, the Pap-to-HPV ratio comprised 6.7:1 in 2025 (Figure 1).

### Bethesda system 2014 category distribution and quality indicators

The data on annual Bethesda category proportions and quality metrics are presented in Table 2 and benchmarked against international standards in Table 3. Across all 5 study years, the reported rates were within the corresponding international benchmark range, with only a small deviation in the unsatisfactory specimen rate in 2024. The 5-year medians were numerically indistinguishable from global published values (Table 3).

**Table 2:** Bethesda System 2014 category proportions and laboratory quality metrics, CSD Laboratory, 2021–2025.

Category/indicator	2021 (pre-war)	2022	2023	2024	2025	Trend *r*	*p*-value
Specimen volumes							
Total Pap tests (*n*)	145,262	114,051	181,709	236,268	269,161	−	−
Year-on-year change (%)	Reference	−21.5%	+59.3%	+30.0%	+13.9%	−	−
Bethesda System 2014 categories (% of informative specimens)							
NILM	90.01	91.61	91.36	89.24	91.27	+0.04	0.955
Non-diagnostic/unsatisfactory (%)	0.54	0.64	0.80	1.03	0.88	+0.918	0.028^a^
Squamous abnormalities—low-grade							
ASC-US	4.56	3.94	4.12	5.38	4.45	+0.323	0.596
LSIL	3.57	2.68	2.83	3.38	2.49	−0.498	0.393
Squamous abnormalities—high-grade							
ASC-H	0.36	0.31	0.21	0.28	0.20	−0.691	0.196
HSIL	0.62	0.52	0.36	0.38	0.31	−0.944	0.016^a^
SCC	0.051	0.032	0.024	0.016	0.023	−0.757	0.139
Glandular abnormalities							
AGC—all subtypes combined^b^	0.247	0.246	0.255	0.232	0.224	−0.210	0.735
AC—all subtypes combined^c^	0.014	0.008	0.004	0.007	0.005	−0.704	0.188
Derived laboratory quality metrics							
Abnormal ≥ ASC-US (%)	9.42	7.74	7.81	9.67	7.70	−	−
ASC/SIL ratio^d^	1.18	1.33	1.36	1.51	1.66	−	−
ASC-US/LSIL ratio	1.28	1.47	1.46	1.60	1.79	−	−
CAP benchmark met: ASC/SIL ≤ 3.0	+	+	+	+	+	−	−
TBS 2014 USR threshold < 1.0% (LBC)	✓	✓	✓	*	✓	−	−

a Statistically significant linear trend (Pearson *r*, *p* < 0.05).

b AGC: AGC-NOS (endocervical, endometrial, glandular) and AGC-FN consolidated.

c AC: invasive adenocarcinoma (endocervical, endometrial, NOS) consolidated.

d ASC/SIL = (ASC-US + ASC-H)/(LSIL + HSIL); CAP upper limit ≤3.0. All proportions expressed as % of informative specimens (total minus non-diagnostic); USR as % of total submissions. 2025 ASC-H corrected from data-entry error (*n* = 517, 0.20%).

*Demonstrates a slight deviation of USR rate compared to benchmark threshold.

**Table 3:** CSD Laboratory Bethesda System 2014 reporting rates (2021–2025) compared with international CAP benchmarks and published global data.

Category	CAP/BS 2014 benchmark (20)	Global data median (range)	CSD 2021–2025 median (range)
Specimen adequacy			
Unsatisfactory (%)	<1.0% LBC	0.80% (0.1–4.5%)	0.80% (0.54–1.03%)
Negative category			
NILM (%)	Expected 85–95%	90.0% (85–94%)	91.27% (89.24–91.61%)
Squamous abnormalities			
ASC-US (%)	3.0–6.0% (LBC)	4.0% (3.0–6.8%)	4.45% (3.94–5.38%)
LSIL (%)	1.5–3.5% (LBC)	2.1% (1.5–3.5%)	2.83% (2.49–3.57%)
ASC-H (%)	<0.4%	0.2% (0.1–0.5%)	0.28% (0.20–0.36%)
HSIL (%)	≥0.3%	0.5% (0.3–0.8%)	0.38% (0.31–0.62%)
SCC (%)	<0.1%	0.030% (0.01–0.07%)	0.024% (0.016–0.051%)
Glandular abnormalities			
AGC all (%)	0.10–0.50%	0.25% (0.10–0.50%)	0.25% (0.22–0.26%)
AC all (%)	<0.05%	<0.05%	0.007% (0.004–0.014%)
Quality metrics			
Abnormal ≥ ASC-US (%)	5–10% (screening)	6.0% (4.0–10.0%)	7.81% (7.70–9.67%)
ASC/SIL ratio	≤3.0 (CAP)	1.4 (0.4–2.5)	1.36 (1.18–1.66)
ASC-US/LSIL ratio	1.0–3.0	1.8 (1.0–3.0)	1.47 (1.28–1.79)

Sources: Eversole et al. (16); Zheng et al. (17).

Naturally, NILM was dominant every year (median 91.27%, range 89.24–91.61% vs. global median 90.0%; no significant trend, *r* = +0.04, *p* = 0.955). The overall abnormal rate (≥ASC-US) declined in 2022 (7.74% vs. 9.42% in 2021; *p* < 0.001), was similar in 2023 (7.81%), rose in 2024 (9.67%, *p* = 0.002), and declined again in 2025 (7.70%, p < 0.001). However, such fluctuations were within the international benchmark range.

ASC-US ranged from 3.94 to 5.38% (median 4.45%, within benchmark 3.0–6.0%). LSIL median comprised 2.83% (range 2.49–3.57%); ASC-H median was 0.28% (range 0.20–0.36%). HSIL remained above the CAP lower quality threshold of 0.3% throughout all years. SCC was rare throughout (median 0.024%, range 0.016–0.051%). AGC (all subtypes consolidated) was stable at a median of 0.25% (range 0.22–0.26%), concordant with global data (17).

Exploring the quality metrics for Pap-test results and interpretation, we found that the ASC/SIL ratio ranged from 1.18 to 1.66 (median 1.36), remaining within the CAP upper limit of 3.0 in all years (global median 1.4). Similarly, ASC-US/LSIL ratio was comparable with global metrics. At the same time, the unsatisfactory rate (USR) showed a significant increasing trend (*r* = +0.918, *p* = 0.028), peaking at 1.03% (*p* = 0.028) in 2024. This small deviation in the unsatisfactory specimens’ rate was followed by a further decline to 0.88% in 2025. The 5-year USR median of 0.80% equals the global published median exactly (16). All values remained within the BS 2014 threshold across all years.

### HPV testing: positivity rates and genotype distribution

HPV test results are shown in Table 4. The overall HPV positivity rate was 21.07% in 2021. It remained statistically comparable during the early wartime years, comprising 19.97% in 2022 and 20.07% in 2023. However, the elevation of HPV testing volumes was associated with a decline in the positivity rate to 17.87% in 2024 (*p* = 0.002) and 17.16% in 2025 (*p* < 0.001 compared to the prewar period). This decline is consistent with a healthy-population dilution effect—as HPV testing expanded significantly, a progressively larger proportion of lower-risk previously unscreened women was reached.

**Table 4:** HPV testing volumes, overall positivity rates, and HPV 16/18 genotype distribution, 2021–2025.

Parameter	2021 (pre-war)	2022	2023	2024	2025	Trend *r*	*p*-value
HPV tests (*n*)	1,452	10,187	24,210	38,762	40,363		
HPV-positive (*n*)	306	2034	4,859	6,869	6,928		
HPV positivity rate (%)	21.07	19.97	20.07	17.72†	17.16^b^	−0.956	0.011^a^
HPV 16/HPV-positive (%)	30.07	28.66	27.91	27.7	32.04	+0.239	0.699
HPV 18/HPV-positive (%)	5.56	7.77	8.48	8.44	10.39†	+0.936	0.019^a^
Other HPV/HPV-positive (%)	64.38	63.57	63.61	63.83	57.56	−0.721	0.169
Pap-to-HPV ratio	100.0:1	11.2:1	7.5:1	6.1:1	6.7:1		

a Statistically significant linear trend (*p* < 0.05).

b Significantly different from 2021 baseline by *χ*^2^ test (*p* < 0.01).

Other HPV: types 31, 33, 35, 39, 45, 51, 52, 56, 58, 59, 66, and 68 combined.

At the same time, when analyzing the demographic composition of the expanding HPV screening cohort, we observe a progressive shift in the age profile of the retrieved cases. The data demonstrated a continuous increase in the proportion of the younger cohort, specifically those aged under 25 and 25–29 years, over the wartime period. The expansion of molecular HPV-testing volumes was accompanied by a highly significant structural shift in the age distribution of the screened population (*p* < 0.001; Supplementary Tables S1, S2). Over the 5-year period, there was a steady increase in the representation of younger cohorts. Specifically, the number of cases in the cohort of women aged under 25 years expanded from 6.82% in 2021 to 9.91 in 2025. Similarly, the shares of cases referred to the age 25–29 years increased from 14.9 to 16.9%. Conversely, the dominant pre-war cohort (aged 30–65 years) showed a slight decline in the overall age-specific structure of the study population, with a relative proportional decline from 77 to 72.6%, demonstrating that the wartime expansion of the state-reimbursed primary HPV screening program successfully mobilized a previously under-screened younger population. This trend in testing was accompanied by a significant change in HPV positivity results among women of various age groups.

Stratification of high-risk HPV positivity rates within different age cohorts revealed distinct, heterogeneous trends over the observed period. The cohort of women under 25 consistently demonstrated the highest burden of high-risk HPV infection. Positivity rates fluctuated without a uniform downward trend, moving from 30.3% in 2021 to a peak of 35.0% in 2024, with further settling at 30.8% in 2025. Similarly, in young adult women aged 25–29 years, HPV positivity remained elevated and relatively stable during the early phase, peaking at 31.5% in 2023. However, a noticeable decline was observed in the later wartime period, dropping to 25.3% in 2024 and 25.1% in 2025. The dominant screening cohort of women aged 30–65 showed a steady, progressive, and highly consistent year-over-year decline in high-risk HPV prevalence. Positivity rates lowered linearly from 18.8% at the 2021 pre-war baseline to 16.7% in 2022, 16.2% in 2023, 13.7% in 2024, and reaching a minimum of 13.5% by 2025. Finally, the cohort over 65 years owned a small absolute test volume, with positivity rates fluctuating, rising from 10.5% in 2021 to a peak of 18.7% in 2023, before declining to 15.2% by 2025.

HPV16 remained the predominant genotype (median 28.7% of positives; with 95% CI 27.5–32.0%) over the observed period. HPV18 showed a statistically significant increasing trend within the cohort of high-risk HPV-positive individuals—from 5.56% of positives in 2021 to 10.39% in 2025 (*p* = 0.019). The proportion of other HPV types (31, 33, 35, 39, 45, 51, 52, 56, 58, 59, 66, 68) ranged from 57.56 to 64.38% without a significant trend.

Assessment of HPV-cytology concordance on a subset of co-tested samples in HPV-tested cases with abnormal cytology revealed that HPV positivity rates rose with cytological severity (Table 5). The rate of HPV-positive results reached 91.0% (95% CI 81.8–96.1%) among HSIL specimens, comprised 62.1% (95% CI 43.0–78.3%) among ASC-H, and was 100% among the two tested SCC cases. Overall, 81 of 98 tested cases (82.7%) were HPV-positive.

**Table 5:** HPV status by the Bethesda cytology category, Q4-2025 case-level subset (HPV-tested cases only, *n* = 98).

Cytology category	HPV+ (*n*)	HPV− (*n*)	HPV tested (*n*)	HPV+ rate among tested (%)	95% CI (%) Wilson score
HSIL	61	6	67	91.0	81.8–96.1
ASC-H	18	11	29	62.1	43.0–78.3
SCC	2	0	2	100.0	34.2–100.0^a^
Total (all categories)	81	17	98	82.7	73.8–89.1

a SCC (*n* = 2): 95% CI calculated by the Wilson score method; interpret with caution due to very small sample.

## Discussion

This 5-year repeated cross-sectional analysis of 946,451 Pap tests and 114,974 HPV tests established three principal findings. First, Ukrainian cervical screening showed quantitative resilience and sustained expansion: following a decline in 2022, Pap test volumes recovered to 85.3% above the pre-war baseline by 2025, while HPV testing expanded sharply. Second, and most clinically significant, all BS 2014 quality indicators fell within international benchmark ranges in every study year, with 5-year medians numerically indistinguishable from global data. Third, HPV 18 proportional share approximately doubled (5.6 to 10.4%, *p* = 0.008), with direct implications for vaccination policy.

The 2022 volume decline is consistent with wartime patterns documented across conflict settings. A previously published systematic review found that armed conflict increases cervical cancer incidence and mortality primarily through infrastructure collapse and resource diversion (5). In Ukraine, war-related impacts included laboratory evacuation, occupation of collection points in frontline regions, mass displacement, and targeted energy infrastructure attacks (6, 19). The COVID-19 pandemic provides the closest methodological comparator. Vigliar et al. (20) documented a 26.5% reduction in cervical cytology volume post-lockdown across 17 countries, with a concurrent increase in malignancy rates. Despite structurally more severe disruption, recovery from the wartime reduction in Ukraine was considerably faster. This paradox is best explained by structural resilience factors, such as institutional protocol continuity and a decentralized collection network of more than 170 regional points of CSD LAB that allowed flexibility (21). In addition, the progressive expansion of screening volumes from 2023 onwards could also be explained by the concurrent expansion of the National Health Services of Ukraine (NHSU) Program of Medical Guarantees (PMG). Based on a “money follows the patient” principle, the NHSU contracts both public and private providers regardless of ownership form. By 2021, NHSU covered 35 specialized care packages, including cancer early detection, and has continued to expand through the wartime period (22). In 2023, CSD LAB entered an NHSU contract, so cervical cytology and HPV testing became available to patients as state-reimbursed services upon receipt of an electronic physician referral. This directly eliminated the financial barrier that had historically suppressed opportunistic screening in Ukraine. Critically, despite the economic and demographic effects of the war, the NHSU maintained universal population coverage under the PMG, enabling continued state-guaranteed access to services across Ukraine (23). Besides, growing population awareness and targeted community outreach are complementary drivers of screening expansion, although our single-laboratory center design does not allow us to specify this contribution. The demonstration that Bethesda categories rate matched international benchmarks across all 5 study years, including those during the acute wartime phase, has not previously been reported for any conflict-affected cytopathology service. The numerical precision of the concordance is important. The CSD 5-year non-diagnostic rate median of 0.80% equals the CAP interlaboratory program global median exactly (16). Important that the ASC/SIL ratio as a quality indicator was also within the CAP upper limit of 3.0 throughout. Although the AGC median of 0.25% matches the global median to two decimal places (17), we did not find a correlation with the rise in HPV-18 proportional shares. This highlights the importance of primary high-risk HPV screening for capturing endocervical glandular precursors.

One of the most striking facts of this study performed in Ukraine is a significant expansion of HPV testing, which is among the most rapid documented in any European setting, achieved during active armed conflict. The described age-specific dynamics demonstrate that while the overall net population positivity rate fell from 21.07 to 17.16% due to massive testing expansion, the true decline was primarily driven by steady clearing and dilution trends within the mature 30–65 age demographic and additional shifts in the cohort of women aged 25–29 years. At the same time, the youngest cohort (under 25 years) maintained persistently high viral pressure. The significant decline in overall HPV positivity reflects population-level dilution as testing reaches a broader, lower-risk population. Such a pattern was documented internationally when organized HPV programs expanded into previously unscreened cohorts (24). The Pap-to-HPV ratio of 6.7:1 in 2025 was still cytology-dominant but narrowed rapidly, indicating a program in transition, consistent with European guidelines recommending primary HPV-based screening (25).

Several factors may contribute to the disproportional expansion of HPV testing relative to cytology during wartime. From a workforce perspective, documented losses of cytopathologists due to war-induced emigration and internal displacement (9, 10) likely created an incentive to shift capacities toward automated molecular platforms that require minimal specialist interpretation. Such an advantage is well recognized in resource-constrained settings (21). From an epidemiological view, the concurrent decline in overall HPV positivity is consistent with a dilution effect as testing reached previously unscreened or under-screened cohorts. This pattern was internationally documented during program scale-up (20). Besides, the implementation of Ukraine’s National Standard on cervical screening in 2024 formally endorsed primary HPV testing driven by WHO and ESGO recommendations (3, 21). Finally, some studies highlight a paradoxical health-seeking effect under armed conflict conditions, as amplified personal vulnerability has been associated with increased engagement with preventive healthcare among people who retained access to services provided through the program of medical guarantees (7). Together, these structural, policy-driven, and behavioral factors provide a plausible multi-level explanation for the observed HPV testing expansion.

The most prominent epidemiological observation was that the youngest cohort (under 25 years) maintained persistently high viral pressure, with high-risk HPV positivity fluctuating between 28.5 and 35.0%. Concurrently, the proportional share of HPV 18 among positive tests nearly doubled, rising from 5.56 to 10.39%. This finding must be interpreted with extreme caution, as it reflects relative genotype distribution dynamics within positive samples rather than true general population prevalence. This proportional shift is highly indicative of an ascertainment effect, occurring as rapid testing expansion successfully captured young cohorts with historically low pre-war vaccine coverage, rather than a true surge in community transmission. Because HPV 18 is disproportionately associated with cervical adenocarcinoma (which is more difficult to detect by cytology alone), this trend highlights the critical importance of primary molecular screening (26). Three potential mechanisms may contribute to this phenomenon. First, wartime geographical redistribution of populations with different genotype exposure histories (eastern regions had higher overall HPV positivity in our regional analysis). Next, ascertainment bias is possible, as HPV testing expansion could reach previously unscreened women with prevalent HPV18 infections. Finally, an epidemiological shift in the context of Ukraine’s very low pre-war HPV vaccination coverage could contribute to the HPV18 positivity trend (27). This observation argues for genotype-specific surveillance in nationally representative cohorts and strengthens the case for accelerating gender-neutral HPV vaccination as a post-conflict health reconstruction priority.

Important that the HPV positivity rate of 91.0% (95% CI 81.8–96.1%) among HSIL specimens is concordant with published benchmarks of 88–95%, confirming that high-grade cytological abnormalities remain overwhelmingly HPV-driven in this population. The substantially lower rate among ASC-H (62.1, 95% CI 43.0–78.3%) is consistent with the known limited positive predictive value of this borderline category (16). Although high-risk HPV negativity was noted in a subset of ASC-H cases, current clinical protocols firmly dictate that a negative molecular result does not rule out the underlying severe disease in this category (3). Immediate colposcopy remains the mandatory standard of care for this high-risk cytological category, regardless of the accompanying molecular testing result.

The results of this study demonstrate that organized, decentralized infrastructure preserves quality under conflict. Completing Ukraine’s transition to a fully organized population-based program with a centralized registry, enabling catch-up screening for women who missed examinations in 2022, would be the most important structural investment.

The demonstrated capacity for multiple-fold HPV test volume expansion under wartime conditions, combined with ESGO/WHO/EU Beating Cancer Plan recommendations, drives the shift toward more active primary HPV-based screening, reducing dependence on the cytologist workforce and extending intervals for HPV-negative women (25). These genotype distribution dynamics provide additional context for informing future immunization strategies. Moving forward, the inclusion of the HPV vaccine in Ukraine’s national immunization calendar represents a promising step toward expanding vaccine access and improving cervical cancer prevention outcomes.

### Limitations of the study

Although this large-scale nation-wide study covered 5 years of observation, there are several limitations that affect the levels of evidence in this study. First, it was a single-center study. Findings reflect a large private urban laboratory and may not represent the practice of state laboratories and/or rural services, where wartime disruptions may have been more severe. In addition, cervical cancer incidence and screening behaviors are heavily stratified by socioeconomic status, which was not addressed in this study. Second, annual data are laboratory-level aggregates precluding patient-level demographic adjustment, exclusion of repeat tests, or calculation of individual screening coverage rates. An additional limitation of this study is the lack of histological follow-up data for patients diagnosed with atypical glandular cells (AGC) or adenocarcinoma (AC). Third, because our data are laboratory-level aggregates, they reflect individual specimen volumes rather than unique patient records. The potential inclusion of repeat tests from the same individuals over the 5-year period may influence the reported cross-sectional HPV positivity rates and annual fluctuations. Next, the HPV-cytology concordance subset is small and based on 98 Pap-HPV-cotested cases in Q4-2025. Estimates for rare categories (SCC *n* = 2) should be interpreted with caution. Finally, there is a geographical accessibility bias as specimens from occupied and frontline regions are absent from the database. The reported rates reflect accessible testing populations rather than the full Ukrainian population. Because CSD LAB operates as an independent diagnostic facility without seamless integration into a unified clinical health registry, it remains difficult to robustly differentiate primary screening from targeted diagnostic follow-up on an individual test level. However, considering the low rates of severe precancerous abnormalities relative to the massive sample size, alongside the visible healthy-population dilution effect in our age-stratified HPV results, the authors believe this cohort represents a predominantly primary screening or re-screening population. Finally, while this study systematically evaluated laboratory testing patterns and cytological quality indicators, downstream clinical and histological outcomes were beyond the scope of the available database and this study. The aggregate dataset lacked linked patient-level data and did not include longitudinal colposcopy referral rates, tissue biopsy results, definitive histological metrics, and the absolute numbers of treated patients undergoing surgical interventions. Future registry-linked or prospective studies are required to track these downstream clinical pathways and fully quantify the long-term impact of wartime screening adaptations on cervical cancer incidence and treatment outcomes in Ukraine.

## Conclusion

Despite 4 years of sustained armed conflict, cervical screening services in Ukraine have expanded substantially beyond pre-war capacity, maintaining full adherence to the Bethesda System and quality standards. Although both cytological and molecular testing increased, the clear shift toward rapid HPV testing expansion was demonstrated. The rise in HPV18 proportional share among HPV-positive cases and declining overall positivity provide important data for planning genotype-specific surveillance and the national HPV vaccination program. This study demonstrated that diagnostic quality and service expansion can be sustained under armed conflict when decentralized infrastructure and institutional resilience are maintained.

## Data Availability

The raw data supporting the conclusions of this article will be made available by the authors, without undue reservation.
