# Supplementary material for: Cervical screening under wartime: resilience and quality of laboratory services in Ukraine during the Russian invasion
Source: Front Public Health. 2026 Aug 11;14:1909774. doi: 10.3389/fpubh.2026.1909774 (PMC13503610; doi:10.3389/fpubh.2026.1909774)
Supplement: Supplementary file 1 [file Table_1.DOCX]

**Table S1. Age distribution of the cases tested for HPV over 2021-2025 period.**

|  | **Under 25** | **25-29** | **30-65** | **Over 65** | **Number by year** |
| --- | --- | --- | --- | --- | --- |
| **Absolute number** | | | | | |
| 2021 | 99 | 216 | 1118 | 19 | 1452 |
| 2022 | 764 | 1589 | 7752 | 82 | 10187 |
| 2023 | 2033 | 3946 | 18038 | 193 | 24210 |
| 2024 | 3570 | 6744 | 28263 | 185 | 38762 |
| 2025 | 4001 | 6821 | 29298 | 243 | 40363 |
| Total | **10467** | **19316** | **84469** | **722** | **114974** |
| **Relative proportion** | | | | | |
| 2021 | 6.82% | 14.9% | 77.0% | 1.31% | 100% |
| 2022 | 7.50% | 15.6% | 76.1% | 0.80% | 100% |
| 2023 | 8.40% | 16.3% | 74.5% | 0.80% | 100% |
| 2024 | 9.21% | 17.4% | 72.9% | 0.48% | 100% |
| 2025 | 9.91% | 16.9% | 72.6% | 0.60% | 100% |
| Mean | **8.37%** | **16.21%** | **74.62%** | **0.80%** |  |

**Table S2. The number and proportion of HPV positive cases among various age groups**

| **Age categories** | **2021** | **2022** | **2023** | **2024** | **2025** |
| --- | --- | --- | --- | --- | --- |
| **Under 2025 (n)** | 30 | 218 | 657 | 1248 | 1232 |
| Proportion (%) | 30.3% | 28.5% | 32.3% | 35.0% | 30.8% |
| **25-29 year (n)** | 64 | 508 | 1244 | 1706 | 1711 |
| Proportion (%) | 29.6% | 28.7% | 31.5% | 25.3% | 25.1% |
| **30-65 years (n)** | 210 | 1294 | 2922 | 3881 | 3948 |
| Proportion (%) | 18.8% | 16.7% | 16.2% | 13.7% | 13.5% |
| **Over 65 year (n)** | 2 | 14 | 36 | 34 | 37 |
| Proportion (%) | 10.5% | 17.1% | 18.7% | 18.4% | 15.2% |
| **Total number of HPV-positive cases** | **306** | **2034** | **4859** | **6869** | **6928** |
| **% of HPV-positivity** | **21.07%** | **19.97%** | **20.07%** | **17.72%** | **17.16%** |
